# Clinical, Radiological, and Genetic Characterization of a Patient with a Novel Homoallelic Loss-of-Function Variant in *DNM1*

**DOI:** 10.3390/genes13122252

**Published:** 2022-11-30

**Authors:** Ruqaiah AlTassan, Hanan AlQudairy, Rakan Alromayan, Abdullah Alfalah, Omar A. AlHarbi, Ana C. González-Álvarez, Stefan T. Arold, Namik Kaya

**Affiliations:** 1 Department of Medical Genomics, Centre for Genomic Medicine, MBC: 75, P.O. Box 3354, King Faisal Specialist Hospital, and Research Centre, Riyadh 11211, Saudi Arabia; 2 College of Medicine, P.O. Box 50927, AlFaisal University, Riyadh 11533, Saudi Arabia; 3 Translational Genomic Department, Centre for Genomic Medicine, MBC: 03, P.O. Box 3354, King Faisal Specialist Hospital and Research Centre, Riyadh 11211, Saudi Arabia; 4 College of Medicine, King Saud University, Riyadh 11451, Saudi Arabia; 5 Department of Radiology, MBC: 28, P.O. Box 3354, King Faisal Specialist Hospital and Research Centre, Riyadh 11211, Saudi Arabia; 6 Bioengineering Program, Biological and Environmental Science and Engineering Division, King Abdullah University of Science and Technology (KAUST), Thuwal 23955-6900, Saudi Arabia; 7 Computational Biology Research Center, King Abdullah University of Science and Technology, Thuwal 23955-6900, Saudi Arabia; 8 Centre de Biologie Structurale (CBS), INSERM, CNRS, Université de Montpellier, F-34090 Montpellier, France

**Keywords:** novel deleterious variant, homozygous, loss of function, *DNM1*, epileptic encephalopathy, intellectual disability, developmental delay, whole-exome sequencing (WES), Sanger sequencing

## Abstract

Heterozygous pathogenic variants in *DNM1* are linked to an autosomal dominant form of epileptic encephalopathy. Recently, homozygous loss-of-function variants in *DNM1* were reported to cause an autosomal recessive form of developmental and epileptic encephalopathy in unrelated patients. Here, we investigated a singleton from a first-degree cousin marriage who presented with facial dysmorphism, global developmental delay, seizure disorder, and nystagmus. To identify the involvement of any likely genetic cause, diagnostic clinical exome sequencing was performed. Comprehensive filtering revealed a single plausible candidate variant in *DNM1*. Sanger sequencing of the trio, the patient, and her parents, confirmed the full segregation of the variant. The variant is a deletion leading to a premature stop codon and is predicted to cause a protein truncation. Structural modeling implicated a complete loss of function of the Dynamin 1 (DNM1). Such mutation is predicted to impair the nucleotide binding, dimer formation, and GTPase activity of DNM1. Our study expands the phenotypic spectrum of pathogenic homozygous loss-of-function variants in *DNM1*.

## 1. Introduction

Developmental and epileptic encephalopathies (DEEs) are a group of neurological disorders characterized by epileptic episodes and progressive cerebral dysfunction [1,2]. These disorders commonly manifest as psychomotor developmental delays, dysmorphic features, or abnormal neurological findings such as hypotonia and intellectual disability [3,4,5,6,7]. Heterozygous pathogenic variants in *DNM1* are linked to autosomal dominant developmental and epileptic encephalopathy, type 31 (DEE31; OMIM: 616346). The clinical hallmark of DEE31 includes global developmental delay and refractory seizures apparent in early infancy. More than 30 individuals have been reported carrying heterozygous variants in *DNM1*, mostly being de novo missense variants with predicted dominant negative effects on highly conserved amino acids in the DNM1.

Patients with DEE31 exhibit various types of refractory seizures in their first months or years of life, which exacerbates their psychomotor deficits and developmental regression. Moreover, the affected patients present with severe to profound intellectual disability, with pronounced hypotonia and absence of speech. Some patients may show additional syndromic features, including dysmorphic features or cortical visual impairment [8].

*DNM1* is located on chromosome 9q34.11 and encodes a GTPase protein, dynamin 1, which is involved in the endocytosis and fission of neurotransmitter vesicles for recycling after an action potential [9]. DNM1 works by self-assembling into a helical polymer around the vesicle membrane and then constricting after GTP hydrolysis, leading to the fission of the vesicle and complete endocytosis [10,11]. Only recently, homozygous deleterious variants were reported in *DNM1* [12]. Here, we present the third homozygous variant in *DNM1* in a female who presented with global developmental delay, myoclonic seizures, facial dysmorphism, and nystagmus.

## 2. Materials and Methods

### 2.1. Patient and Sample Collection

**Figure 1:**
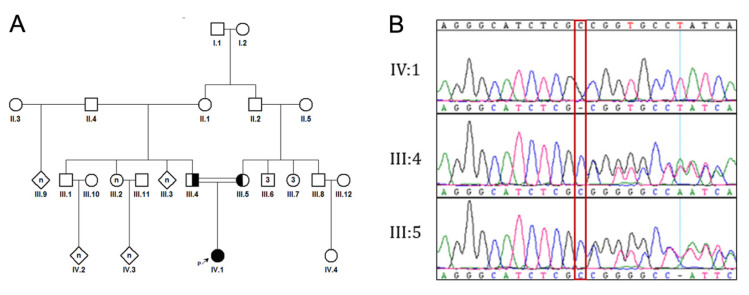
Genetic Analysis. (**A**) Pedigree of the family 1 showing the carrier parents, the affected girl and the consanguineous marriage. The affected girl is labeled with a filled symbol (black colored). The carrier parents are displayed with half-filled symbols. Squares indicates male individuals, whereas circles show females. (**B**) Sanger sequencing results of the variant are shown in chromatograms. The red box shows the deletion in IV:1.

The patient was clinically evaluated at the Medical Genetics clinic at King Faisal Specialist Hospital and Research Centre, Saudi Arabia. Peripheral blood was taken from the patient and her parents (first-degree cousins, Figure 1A) in EDTA tubes for testing, after obtaining their consent.

### 2.2. DNA Isolation, Molecular Study

The blood (3 milliliters) was collected from the patient and her parents in EDTA tubes. DNA extraction was performed using the Gentra^®^ Puregene DNA Purification Kit according to the manufacturer’s instructions (Gentra Systems, Inc. Minneapolis, MN, USA). The quantity and quality of the extracted DNA were subsequently evaluated using a NanoDrop^®^ ND-1000 (NanoDrop Inc., Wilmington, DE, USA). Clinical exome sequencing was performed on genomic DNA. A library was prepared using the DNA, which was enzymatically fragmented. DNA capture probes were used to enrich the human coding exome and mitochondrial genome. The generated library was sequenced using an Illumina platform to obtain at least 20× coverage depth. Following alignment, variant calling, and annotation, the generated VCF files underwent a thorough filtration procedure utilizing national and international databases. All variants (minor allele frequency, MAF: <1%) in the gnomAD database and the disease-causing variants reported in commercially available, such as HGMD^®^, and publicly accessible, i.e., ClinVar and OMIM, databases were searched. The process included variants in coding exons and exon/intron boundaries (±10 bases of intronic sequences). All patterns of inheritance were taken into consideration. Moreover, family history and clinical information were also used to evaluate the identified variants with respect to their pathogenicity and causality. 

**Figure 2:**
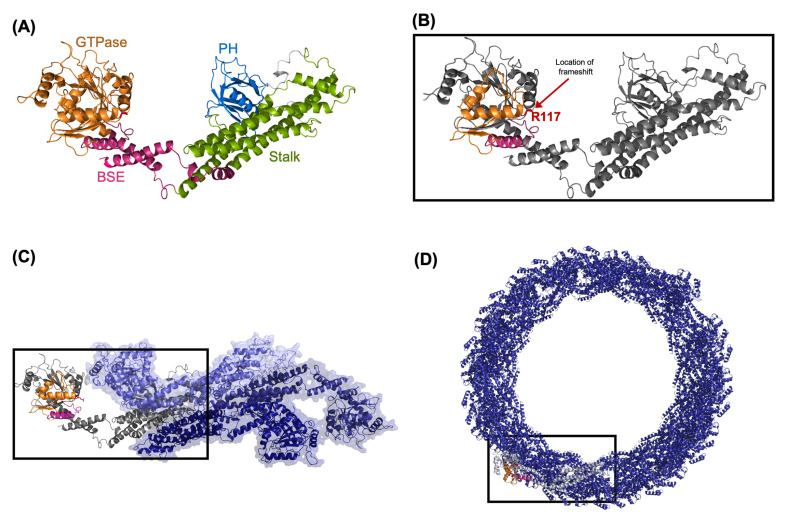
Molecular environment and effect of the Pro 117 Arg fs*14 mutation on the GTPase domain. (**A**) Cartoon diagram of dynamin 1 based on the crystal structure of dynamin 3 (PDB 5A3F) with the GTPase domain shown in orange, the bundle signaling element (BSE) shown in pink, the stalk domain shown in green, and the pleckstrin homology (PH) domain shown in blue. (**B**) Diagram of the effect of the Pro117Argfs*14 mutation, with the deleted portion of the protein highlighted in gray. The substituted Proline (R117) is shown as a red stick. (**C**) Location of the variant within the homology model of tetrameric dynamin 1 based on the crystal structure of dynamin 3 (PDB 5A3F). (**D**) Location of the variant within the cryo-EM structure of multimeric dynamin 1 (PDB 7AX3).

Specific primers flanking the targeted region were designed using the Primer 3 web tool. PCR was optimized using human control DNA. Afterward, DNA was amplified by PCR and sequenced using Sanger sequencing (Figure 1B). The dynamin 1 sequence was retrieved from the Uniprot database. A model for amino acids #6-791 was produced with SWISS-MODEL [13], based on the homologous crystal structure of dynamin 3 (PDB ID 5A3F, identity = 84.46) as a template. Modeling of dynamin 1 resulted in a homo-tetramer structure (Figure 2A,C). The location of the variant was also studied within the cryo-EM structure of multimeric dynamin 1 (PDB 7AX3) (Figure 2B,D). The models were manually inspected, and the variants were evaluated using the Pymol program (pymol.org) accessed on 5 October 2022.

## 3. Results

### 3.1. Clinical Features

**Figure 3:**
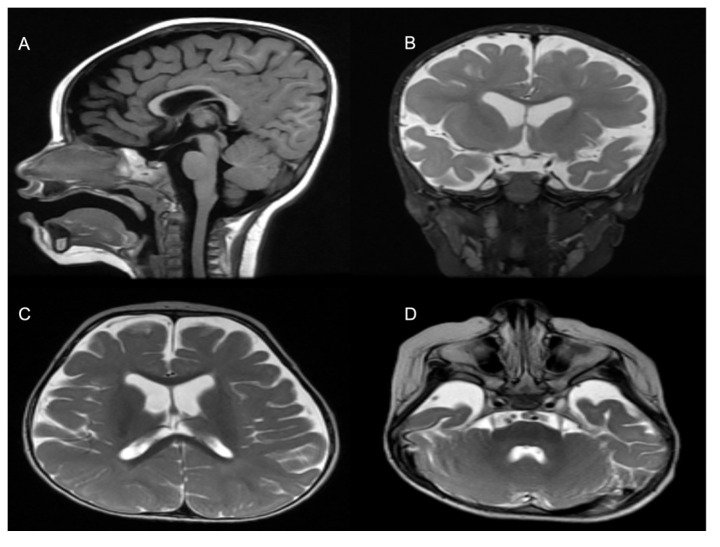
MRI Findings. (**A**) Mild thinning of the corpus callosum. Selected coronal T2 (**B**) and axial T2-weighted images (**C**,**D**) show volume loss of the cerebral hemispheres with resultant mild expansion of the lateral ventricles and CSF spaces.

The patient was generated by in vitro fertilization from healthy consanguineous parents with a history of infertility. She was born at term after an uneventful pregnancy, with a normal birth weight. The patient started showing symptoms at 4 months of age, when she was not able to support her head, roll over, make sounds, or follow subjects. She failed to gain any of her developmental milestones including gross and fine motor function, cognition, and speech. She had multiple choking episodes, mainly with liquids and only tolerated a pure diet. She had constipation. Her first attack of seizure was at the age of 16 months, after a febrile illness, and she continued to suffer multiple brief myoclonic seizures, which were partially controlled with levetiracetam. Assessment at the age of 18 months was remarkable for facial dysmorphism in the form of bifrontal narrowing, epicanthal fold, almond-shaped eyes, long palpebral fissures, low-set ears, protruded tongue, and nystagmus. The growth parameters were within the percentiles for age and gender (head circumference 46 cm (25th percentile), weight 9 kg (10th percentile), and length 90 cm (>90th percentile). Neurological deficit was evident, with central hypotonia, peripheral limbs spasticity, hyperreflexia, and positive clonus. An ophthalmological exam was positive for moderate rod–cone dystrophy. Brain magnetic resonance imaging (MRI) at the age of 12 months showed a global mild volume loss of the cerebral hemispheres with a resultant mild expansion of the lateral ventricles and CSF spaces, a mildly thin but well-formed corpus callosum, a normal volume and appearance of the brainstem and cerebellum, and appropriate myelination (based on the patient’s age). Moreover, the brain showed normal signal characteristics without cortical, structural, or migration abnormalities (Figure 3). Hearing assessment was normal. 

### 3.2. Genetic Findings

The diagnostic clinical exome sequencing showed a homozygous deletion in *DNM1* (NM_001288739.1: c.350del, p.Pro117Argfs*14). The variant is a single-base deletion that creates a shift in the reading frame (codon 117) and causes a truncation at a new stop codon at the 13th position downstream (p.Pro117Argfs*14). Confirmatory Sanger sequencing indicated the presence of the deletion in the patient, and parental testing confirmed the in trans inheritance.

### 3.3. Variant’s Classification

The variant was classified as pathogenic according to the standards and guidelines for the interpretation of sequence variants of the American College of Medical Genetics (ACMG) based on the following criteria: PVS1 (Very strong evidence: Null variant (frameshift) in DNM1), predicted to cause NMD (nonsense-mediated decay), predicted to cause a disease outcome based on in silico classifiers and structural analysis, and PM2 (Strong evidence: absent in gnomAD exomes and gnomAD).

### 3.4. Structural Analysis of the DNM1 Variant and Predicted Functional Effect

Dynamin 1 contains five distinct domains: an N-terminal GTPase (G) domain mediating nucleotide binding and hydrolysis, a bundle signaling element (BSE), a stalk, a pleckstrin homology (PH) domain involved in lipid binding, and a proline-rich domain (PRD) mediating interactions with scaffolding proteins containing BAR and SH3 domains. The variant is a deletion leading to a premature stop codon. The Pro117Argfs*14 mutation results in a truncation that leads to a complete loss of function of the GTPase domain. Hence, the mutation would impair the nucleotide binding, dimer formation, and GTPase activity of the G domain. Because GTP hydrolysis is necessary for vesicle scission, disruptions to this process lead to an inefficient recycling of synaptic vesicles, with impaired tonic firing at inhibitory synapses and thus seizures.

## 4. Discussion

DNM1 involves in the endocytosis and fission of neurotransmitter vesicles for recycling, works by self-assembling into a helical polymer around the vesicle membrane, and provides the mechanical force necessary to pinch off budding vesicles from the synaptic membrane [14]. Hence, it has essential function during intense neuronal activity. The expression of *DNM1* mRNA is upregulated during postnatal brain development and peaks during neurite and synapse formation. During receptor-mediated endocytosis, dynamin molecules assemble into tetramers that hydrolyze GTP [14]. Upon GTP hydrolysis, DNM1 oligomerizes at the neck of clathrin-coated vesicles to mediate constriction and subsequent scission of the membrane [15]. Structural and physiological analysis of the resulting transcribed proteins indicate a dominant-negative effect that leads to impairments in GTP hydrolysis. Such affect eventually causes impaired vesicle endocytosis, which adds credence to the notion that impaired recycling of the vesicle is a key step in epileptogenesis [11]. Of note, nearly one-third of the affected individuals present the recurrent p.Arg237Trp mutation, which affects an arginine residue within the GTPase domain; the majority of the reported deleterious variants are located in the GTPase and middle domains, both of which are considered functionally critical [8]. 

In this study, we presented another autosomal recessive case caused by a novel deleterious homozygous loss-of-function variant in *DNM1* and provided detailed clinical, neuroradiological, and molecular results of a patient who presented with developmental and epileptic encephalopathy. 

Since the first case of a pathogenic *DNM1* variant was discovered nearly a decade ago, most of the cases were reported to be in autosomal dominant mode, caused by heterozygous deleterious variants. The only exception to this was the study describing two unrelated DEE cases harboring loss-of-function nonsense variants in a homozygous state [12]. Interestingly, both nonsense variants in Yigit et al. (2022) study caused an immediate stop codons (c.97C>T; p.Gln33* in family 1, and c.850C>T; p.Gln284* in family 2) in *DNM1* which led to an early truncation of DNM1. Both patients presented with a severe neurodevelopmental phenotype in the form of hypotonia, visual impairment, uncontrolled seizure, and microcephaly, in addition to agenesis of the corpus callosum in one of them. Our patient presented with a global developmental delay, severe hypotonia, notable hyperreflexia, nystagmus, and facial dysmorphism. Moreover, the neuroimaging of our patient showed a global mild volume loss in the cerebral hemispheres with a resultant mild expansion of the lateral ventricles and CSF spaces, in addition to mild agenesis of the corpus callosum, but the rest of the brain was mostly unremarkable, in comparison to Yigit et al. (2022) cases [12]. Interestingly, the novel deletion also resides in the GTPase domain, similar to the other loss-of-function variants reported before [8]. 

The clinical features observed in our patient are similar to those reported for the other two patients; most prominently, global developmental delay, epilepsy, and the hypotonia. Furthermore, it is worth to note that our patient had an additional phenotype including notable hyperreflexia and lower limbs spasticity, with a multitude of dysmorphic features such as bifrontal narrowing, epicanthal folds, low-set ears, nystagmus, and unusual tongue protrusion, a feature being unique among the reported recessive cases. Table 1 summarizes the clinical phenotypes of our patient and of the previously reported patients with homozygous variants in *DNM1*.

To confirm our findings, the genetically tested parents were interviewed and examined. They were asymptomatic and free from any exhibited signs or symptoms of DEE. They also did not show any genotypic variation in the *DNM1* further exemplifying the autosomal recessive mode of transmission for the variant. Of note, as previously reported before, heterozygous knockout mice [16] as well as heterozygous carriers [12] are all normal, similar to the parents (heterozygous carriers) reported in our study. Therefore, as emphasized by Yigit et al. (2022), haploinsufficiency does not involve in the pathobiological mechanism of DNM1-associated DEE in humans.

## 5. Conclusions

In conclusion, we provide further evidence that deleterious homozygous loss-of-function variants in *DNM1* can lead to severe neurodevelopmental defects characterized by epilepsy, global developmental delay, and hypotonia.

## Figures and Tables

**Table 1:** Clinical features of patients with a homoallelic loss-of-function variant in *DNM1*.

Reference		This Study	Yigit et al. (2022)	
Family #		1	2	3
Subjects		1	1	2
Gender		Female	Female	Female
Age at the presentation		4 months	15 weeks	-
Current age		14 months	5 years	-
Consanguinity		(+)	(+)	(+)
Ethnicity		Arab	Arab	Arab
variant	cDNA change	C.350del	c.97C>T	c.850C>T
	Amino acid change	p.Pro117Argfs*14	p.Gln33*	p.Gln284*
	Zygosity	Homozygous	Homozygous	Homozygous
Neurological findings		Seizures (Myoclonic)	Seizures (Myoclonic)	Seizures (Myoclonic, tonic)
		Global developmental delay (cognition, motor, speech) Central hypotonia Hyperreflexia and clonus Spasticity	Muscular hypotonia Poor visual fixation Distal limb dystonia Opisthotonus	Muscular hypotonia Poor visual fixation Speech problems
Neuroimaging		Mild thinning of the corpus callosum Global mild volume loss of the cerebral hemispheres Mild expansion of the lateral ventricles and CSF spaces	Thin corpus callosum Colpocephaly	Mildly dilated ventricles and subarachnoid spaces Widened perisylvian fissure Frontal brain atrophy
EEG		Normal	Hypsarrythmia slow background multifocal spikes	Hypsarrythmia
Genitourinary		Normal	Normal	Normal
GI problems		Constipation, feeding difficulties with liquids	-	Feeding difficulties
Ophthalmological		Nystagmus moderate con-rod dystrophy	Poor visualization	Poor visualization
Dysmorphic features		Bifrontal narrowing, epicanthal folds almond shaped eyes low set ears, protruded tongue	None	Gingival hypertrophy
Hearing		Normal	-	-

## Data Availability

Not applicable.
